# Alleviation of Kainic Acid-Induced Brain Barrier Dysfunction by 4-O-Methylhonokiol in *In Vitro* and *In Vivo* Models

**DOI:** 10.1155/2015/893163

**Published:** 2015-01-20

**Authors:** Jin-Yi Han, Sun-Young Ahn, Jae Hyeon Yoo, Sang-Yoon Nam, Jin Tae Hong, Ki-Wan Oh

**Affiliations:** ^1^Research Institute of Veterinary Medicine, Chungbuk National University, Cheongju 361-763, Republic of Korea; ^2^Lee's Biotech Co., Korea Research Institute of Bioscience and Biotechnology, Daejeon 305-606, Republic of Korea; ^3^College of Pharmacy, Chungbuk National University, Cheongju 361-763, Republic of Korea; ^4^College of Veterinary Medicine, Chungbuk National University, Cheongju 361-763, Republic of Korea

## Abstract

This experiment was designed to investigate whether 4-O-methylhonokiol (MH), a principal ingredient of *Magnolia (M.) officinalis* bark, alleviated acute intraperitoneal (i.p.) kainic acid- (KA-) induced brain blood barrier dysfunction (BBBD) *via* pathological examination and cytological analyses of the brain tissues of mice. KA (10–30 mg/kg) time- and dose-dependently increased the water content of brain tissues and induced edema and encephalopathy. However, pretreatment with MH (5 and 20 mg/kg, i.p.) significantly reduced the water content of the brain compared to that observed in the KA control group. Furthermore, MH significantly and dose-dependently reversed the remarkable variations in evan's blue dye (EBD) staining and malondialdehyde (MDA) levels that were induced by KA (10 mg/kg, i.p.). MH also decreased the elevated seizure scores that were induced by KA (10 mg/kg, i.p.) in mice in a manner similar to scavengers such as DMTU and trolox. Additionally, MH significantly scavenged intracellular ROS and Ca^2+^ within hippocampal cells. The tight junction seals mediated by claudin (Cld-5) were also found to be modulated by MH. MH efficiently reduced 1,1-diphenyl-2-picrylhydrazyl (DPPH) (IC_50_, 52.4 mM) and ^•^OH with an electron spin resonance (ESR) signal rate constant of 4 × 10^9^ M^−1^ · S^−1^, which is close to the reactivity of the vitamin E analog trolox. Taken together, these results suggest that MH may enhance radical scavenging in lipid and hydrophobic environments, which may be important for the physiological activity of the barrier.

## 1. Introduction

2-[4-Methoxy-3-(2-propenyl)phenyl]-4-(2-propenyl)phenol(MH) is a principal ingredient of the bark of* Magnolia (M.) officinalis. Magnolia *bark has been used in traditional medicine to treat various disorders [1, 2]. MH has potent antifungal and antibacterial activities and apparently also has anti-inflammatory and neurotrophic activities [3–5]. MH has a biphenyl (ortho- and parapositions) structure containing two allyl groups, which are beneficial for increasing its affinity for endothelial cells [6]. The ortho-allyl group may potentially form a six-member ring after absorption of the hydroxyl group, but a single para-allyl group cannot form a six-member ring with hydroxyls. Honokiol has been found to be a potent scavenger of hydroxyl radicals due to its allyl groups [6] or phenolic constituents [7, 8]. Phenolic compounds have strong free radical scavenging activities [9, 10]. Additionally, the methylation in the structure of phenolic compounds reduces their overall hydrogen bonding potential [11]. This reduction in hydrogen bonding potential increases the lipophilicity or membrane permeability of these compounds [11]. Lin et al., reported that honokiol possesses the abilities to block excitatory amino acid- (EAAs-) evoked cation signals [12] and to inhibit glutamate-induced cell damage [13]. Moreover, honokiol has antinociceptive actions in glutamatergic pain [14] and antioxidants that suppress the oxidation of low-density lipoproteins due to its strong radical scavenging activities [15].

Electron spin resonance (ESR) is a sophisticated spectroscopic technique that detects free radicals or inorganic complexes in chemical and biological systems [16–18]. ESR spectroscopy of spin-trapped radicals has become the method of choice for the detection and identification of the free radicals that are formed in biological systems [19–21]. The spin-trapping technique utilizing nitrones has been applied to the detection of free radicals for over thirty years. Nitrone spin traps are used in ESR studies because they specifically react with free radicals to form a radical adduct with a longer lifetime than the initial free radicals. For biological applications, nitrone spin traps, such as 5,5-dimethyl-l-pyrroline-*N* oxide (DMPO), have been used most frequently.

EAAs, such as glutamate, are acknowledged as the primary neurotransmitters that mediate synaptic excitation in the vertebrate central nervous system (CNS) [22]. Glutamate has dual actions on CNS neurons; glutamate acts as an excitatory neurotransmitter at physiologic concentrations and acts as a neurotoxic substance when present in excessive amounts. Glutamate has also been implicated in the initiation of nerve cell death in stroke, epilepsy, and other forms of CNS insult. Moreover, glutamate kills neuronal cells through either the receptor-mediated pathway or* via *the inhibition of cysteine uptake and the oxidative pathway [23]. It is well known that KA, a glutamate analogue, induces elevations in intracellular Ca^2+^ and extracellular glutamate levels* via *the coactivation of NMDA receptors. Glutamate-evoked Na^+^ influx has also been proposed to contribute to acute forms of neurotoxicity [24, 25]. Brain blood barrier dysfunction (BBBD) has been described after KA administration [26, 27].

The brain blood barrier (BBB) is a vascular system that regulates the passage of materials between the peripheral circulation and the CNS. The BBB is a unique membranous barrier that tightly segregates the circulating blood [28, 29]. Moreover, the BBB is extremely important “guardian” that regulates the access of drugs to the CNS in both physiological and pathological circumstances [30]. Many CNS diseases and injuries, such as epilepsy, stroke, multiple sclerosis (MS), encephalomyelitis (EAE), and others, are accompanied by BBBD [31–34]. Moreover, barrier dysfunction related to faulty out-transport of metabolites may be responsible for epileptogenesis in the presence (or absence) of other parenchymal abnormalities [10, 35]. The permeability of the endothelial barrier is regulated by two different routes: the paracellular pathway, which controls the permeability through interendothelial junctions, and the transcellular pathway, which involves caveolae-mediated vesicular transport [36]. BBBD and subsequent edema are major contributors to the pathogenesis of epilepsy. The formation of brain edemas has been linked to the inability of the barrier to maintain the necessary ion gradients. Furthermore, alterations in barrier ion homeostasis have been linked to epilepsy [37, 38]. In this study, we examined the therapeutic effects of MH in KA-induced brain barrier dysfunction that are mediated through redox repair.

## 2. Materials and Methods

### 2.1. Chemicals

**Figure 1:**
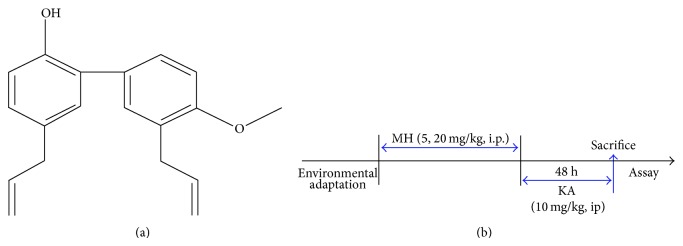
Chemical structure of MH (a) and experimental scheme (b).

MH (molecular weight = 280.4, molecular formula = C_19_H_20_O_2_), the chemical structure of which is shown in Figure 1(a), was isolated from the bark of* M. officinalis* as described elsewhere [39]. The bark of* M. officinalis* was dried in the shade at room temperature and stored in a dark, cold place until use. The air-dried bark of* M. officinalis* (3.0 kg) was cut into pieces and extracted twice with 95% (v/v) ethanol (by weight, four times as much ethanol as dried plant was used) for 3 days at room temperature. After filtration through 400-mesh filter cloth, the filtrate was refiltered through filter paper (Whatman, number 5) and concentrated under reduced pressure. The extract (450 g) was then suspended in distilled water, and the aqueous suspension was extracted with n-hexane, ethyl acetate, and n-butanol. The n-hexane layer was evaporated, and the residue (70 g) was measured chromatographically on silica gel with an n-hexane : ethyl acetate (9 : 1) solution to extract a crude fraction that included MH. This fraction was repeatedly purified by silica gel chromatography using n-hexane : ethyl acetate as the eluent to obtain pure MH (Figure 1). The purity exceeded more than 99.5%. MH was identified by 1H NMR (400 MHz, CdCl_3_) 1 : 3.36 (2H, d, *J* = 7 Hz, H-7), 3.44 (2H, d, *J* = 7 Hz, 7-H), 3.89 (3H, s, OMe), 5.05–5.14 (5H, m, H-9, H-9, OH), 5.93–6.07 (2H, m, H-8, H-8), 6.92 (1H, d, *J* = 7 Hz, Ar-H), 6.97 (1H, d, *J* = 8 Hz, Ar-H), 7.04–7.08 (2H, m, Ar-H), 7.24–7.31 (2H, m, Ar-H). 13C NMR (100 MHz, CDCl_3_) 1 : 34.5 (C-7), 39.6 (C-7), 55.8 (OMe), 111.2 (C-3), 115.7 (C-4), 115.8 (C-9), 116.1 (C-9), 128.0 (C-1), 128.1 (C-6), 129.0 (C-3), 129.2 (C-1), 130.0 (C-5), 130.4 (C-6), 130.7 (C-2), 132.4 (C-5), 136.7 (C-8), 138.0 (C-8), 151.0 (C-2), and 157.2 (C-4). The ethanol extract of* M. officinalis* was composed of 16.6% MH, 16.5% honokiol, 12.9% magnolol, and 42–45% other components. These results agree with previously published data [40], and this compound may possibly be the same compound demonstrated by Zhou et al. [41]. MH was kindly provided by the Bioland Cooperation (Daejeon, South Korea). KA ((2*S*,3*S*,4*S*)-carboxy-4-(1-methylethenyl)-3-pyrrolidineacetic acid) was purchased from Tocris (Ellisville, MO, USA). Cld-5 was purchased from Abcam Inc. (Cambridge, MA, USA). The OXYTEK thiobarbituric acid reacting substances (TBARS) assay kit was purchased from Alexis (Farmingdale, NY, USA), and 6-carboxy-2′,7′-dichlorofluorescein diacetate (DCFH-DA) and fura-4/AM were purchased from Molecular Probes Inc. (Eugene, OR, USA). DMPO was purchased from Enzo (Plymouth Meeting, PA, USA). Evan's blue dye (EBD), Na_2_SO_4_, acetone, ferrous sulfate (Fe_2_SO_4_ *·*7H_2_O), hydrogen peroxide (H_2_O_2_, 30%), diethylenetriamene pentaacetate (DTPA), 1,1-diphenyl-2-picrylhydrazyl (DPPH), 6-hydroxy-2,5,7,7-tetramethylchroman-2-carboxylic acid (trolox),* N*,*N*′-dimethylthiourea (DMTU), 3-(4,5-dimethylthiazol-2-yl)-2,5-diphenyl tetrazolium bromide (MTT), and all other chemicals were of high quality and were obtained from Sigma (St. Louis, MO, USA).

### 2.2. Animals

Male ICR mice (Samtako, Osan, Korea) weighing 30–35 g were used for the* in vivo* experiments (*n* = 7-8). The animals were housed in acrylic cages (45 cm × 60 cm × 25 cm) with water and food available* ad libitum* under an artificial 12 h light/dark cycle (lights on at 7:00) and constant temperature (22 ± 2°C). The mice were housed in a departmental room for 1 week prior to testing to ensure that they had adapted to the new environment. All experiments involving animals were performed in accordance with the National Institutes of Health Guide for the Care and Use of Laboratory Animals (NIH publication number 85-23, revised 1985). The institutional animal care and use committee of Chungbuk National University approved the protocol.

### 2.3. Primary Hippocampal Neuronal Cell Culture and KA Exposure

Primary cultures of rat hippocampal neurons were prepared from the hippocampi of E18-19 Sprague Dawley (SD) rat embryos and cultured according to a previously described method [42]. The hippocampi were dissected and incubated with 0.25% papain in Ca^2+^- and Mg^2+^-free Hank's balanced salt solution at 37°C for 20 min. The cells were then mechanically dissociated with fire-polished Pasteur pipettes by trituration and plated on poly-L-lysine-coated cover slips in a 35 mm culture dish. The cells were maintained in neurobasal/B27 medium containing 0.5 mM L-glutamine, 25 *μ*M glutamate, 25 *μ*M 2-mercaptoethanol, 100 U/mL penicillin, and 100 *μ*g/mL streptomycin under a humidified atmosphere of 95% air and 5% CO_2_ at 37°C, half of the medium was changed every 2 days, and the hippocampal neurons were cultured for 12–14 days before KA (100 *μ*M) exposure. MH (30–100 *μ*M) was added 0.5–1 h prior to KA treatments.

### 2.4. Cell Viability Assays

Cell viability assays were performed as previously described [43]. After exposure for the indicated times, the neurons were assayed for viability using MTT (Sigma, St. Louis, MO, USA), which was added at a final concentration of 5.0 mg/mL for 4 h. MTT was then removed, and the neurons were lysed in 200 *μ*L of dimethyl sulfoxide (DMSO). Absorbance was measured at 570 nm on a SpectraMax M2 multimode microplate reader (Sunnyvale, CA, USA). The data are expressed as the percentages of unexposed neurons that remained in the presence of KA.

### 2.5. Intracellular ROS Measurement

The production of ROS in the neurons was determined as previously described using DCFH-DA (Molecular Probes, Eugene, OR, USA) [44]. The cells were incubated with 10 *μ*M DCFH-DA at 37°C for 30 min. After the DCFH-DA was removed, the cells were recorded. The DCFH-DA-loaded cells were placed in a SpectraMax M2 multiwell fluorescence microplate reader (Sunnyvale, CA, USA) with excitation and emission wavelengths of 515 nm and 552 nm, respectively. The protein concentrations were determined with Bradford assays.

### 2.6. Intracellular Calcium Measurement

The acetoxymethylester form of fura-4 (Molecular probes, Eugene, OR) was used as the fluorescent Ca^2+^ indicator. The hippocampal cells were incubated for 60 min at room temperature with 5 *μ*M fura-4/AM and 0.001% Pluronic F-127 in a HEPES-buffered solution composed of the following (in mM): 150 NaCl, 5 KCl, 1 MgCl_2_, 2 CaCl_2_, 10 HEPES, and 10 glucose with the pH adjusted to 7.4 with NaOH. The cells were then washed with HEPES-buffered solution and placed on a SpectraMax M2 multiwell fluorescence microplate reader (Sunnyvale, CA, USA). The emitted fluorescence was calculated using a fluorescence analyzer and converted to intracellular free Ca^2+^ concentration [Ca^2+^]_*i*_.

### 2.7. Determination of Brain Edema

Water content measurements of the brains of mice that were sacrificed 48 h after the administration of KA (i.p.) were performed. A control group of mice received saline. The water contents of the brain tissues were detected by measuring the ratios of the body weights and brain weights. Briefly, the brains (*n* = 5-6) were quickly removed and weighed (brain weight), and the percentages of water content were calculated as [brain weight/bodyweight] × 100%.

### 2.8. Evaluation of Brain Barrier Integrity

A variety of studies have used the peripheral injection of Evan's blue dye (EBD) as a barrier tracer marker [45]. To estimate barrier integrity, the mice were intravenously injected with EBD (1%, Sigma, St. Louis, MO, USA) in phosphate-buffered saline (PBS, pH 7.4) that had been sterilized by passage through a Millex-GP0 22 *μ*m filter (Millipore, Bedford, MA, USA). Five minutes later, KA (10–30 mg/kg, i.p.) was administered to the mouse. MH was injected into the abdominal site 50 min prior to EBD. Two days later, the mouse was killed by cervical dislocation and the brain was excised. Approximately 1.0 g of brain was minced and dispersed in 6.0 mL 0.5% Na_2_SO_4_ solution, and the dye was extracted by the addition of 14 mL acetone. After 3.5 h of extraction, the dye concentration was determined using a spectrophotometer at OD_590_. MH (5–20 mg/kg, i.p.), DMTU (50 mg/kg, i.p.), and trolox (50 mg/kg, i.p.) were administered 50 min prior to EBD injection.

### 2.9. Measurement of Seizure Activity

Male mice were grouped (*n* = 5 or 6 mice/group) and pretreated (i.p. injection) with MH (5–20 mg/kg) or NaCl (0.9%). Seizures were induced in mice in the KA and MH + KA groups* via *the injection of KA (10–30 mg/kg, i.p.). The mice in the control group received an equal volume of 0.9% NaCl at the same time points. The mice were pretreated (i.p.) with MH and scavengers such as trolox (50 mg/kg, i.p.) and DMTU (50 mg/kg, i.p.) 50 min prior to the KA injections. Male ICR mice weighing 30–35 g were injected with saline or KA dissolved in saline (10–30 mg/kg, i.p.). Seizure activity was rated during a 3 h period after the KA challenge according to the following scale devised by Racine et al. [46]: stage 1 (facial clonus), stage 2 (nodding), stage 3 (forelimb clonus), stage 4 (forelimb clonus with rearing), and stage 5 (rearing, jumping, and falling). Two days after the KA treatment, the hippocampi of the other seizing mice were dissected to evaluate the dysfunctions.

### 2.10. Lipid Peroxidation Assay

Lipid peroxide formation was analyzed by measuring the TBARS in the homogenates as described by Suematsu et al. [47]. The OXYTEK TBARS assay kit was used for these measurements. Lipid peroxidation was determined using the protocol of these authors* via* the measurement of the absorbance at 532 nm and is expressed as nmol of malondialdehyde (MDA)/mg of protein. The protein concentrations of the hippocampi were determined using the Bradford assay.

### 2.11. Western Blotting Assay

Tissues were lysed in the lysis buffer for 30 min on ice with vortexing every 5 min. The lysates were then centrifuged at 14,000 rpm for 5 min to remove the insoluble material. Protein concentrations were determined by the Bradford method (Bio-Rad) using BSA as the standard. For Cld-5, the protein was separated on 16% SDS-PAGE gels. The gels were subsequently transferred onto PVDF membranes (Amersham Hybond TM-P, GE Healthcare, Buckinghamshire, UK) by electroblotting for 2 h at 60–75 V. The membranes were then blocked with 5% nonfat milk solution in Tris-NaCl buffer (TNT) containing 0.5% Tween-20 and incubated with primary antibodies as indicated. Monoclonal donkey anti-rabbit IgG horseradish peroxidase-conjugated secondary antibodies were used at 1 : 3,000. Proteins were detected by enhanced chemiluminescence using a commercial kit (Amersham Hybond TM-P, GE Healthcare, Buckinghamshire, UK).

### 2.12. DPPH Assay

The scavenging of the stable free radical DPPH by MH was assayed spectrophotometrically [48]. DPPH in ethanol (0.1 mM, control) was mixed thoroughly with various concentrations of MH (1–100 mM), and the absorbance was read at 517 nm. The degree of DPPH radical scavenging activity of MH was calculated as a percentage of inhibition (% inhibition) where

(1) % inhibition=Acontrol−AsampleAcontrol×100.

### 2.13. ^•^OH Scavenging Activity by ESR

^•^OH was generated by the Fenton Reaction System, and the generated ^•^OH rapidly reacted with the nitrone spin trap DMPO [21]. The resultant DMPO/^•^OH adduct was detected with an ESR spectrometer. MH (0.2 mL) at various concentrations was mixed with DMPO (0.2 M, 0.2 mL), Fe_2_SO_4_ (2.0 mM, 0.2 mL), and H_2_O_2_ (2.0 mM, 0.2 mL) in a phosphate buffer solution (100 mM, pH 7.2), and the mixture was transferred to a quartz flat cell for ESR measurement. The measurements were performed in an ESR cavity at room temperature (24-25°C). After the reaction, the ESR spectrum was recorded at room temperature using an ESR (JEOL JESTE-300) spectrometer (JEOL, Inc., Tokyo, Japan) equipped with a TE102 cavity. The experimental conditions were as follows: magnetic field, 339.3 ± 10 mT; power, 2.2 mW; modulation frequency, 9.44 GHz; amplitude, 10 × 10; and sweep time, 0.5 min. The results are indicated as the time required to produce a 50% inhibition or as the decrease in signal peak height (IH_50_) by ESR.

### 2.14. Statistical Analyses

The data are presented as the means ± the SEMs. The statistical comparisons were made using one-way analyses of variance (ANOVAs). *P*  values < 0.05 were considered statistically significant. When significant variations were found, the individual values were compared with Holm-Sidak tests.

## 3. Results

### 3.1. Effects of MH on KA-Induced Brain Edema

**Figure 2:**
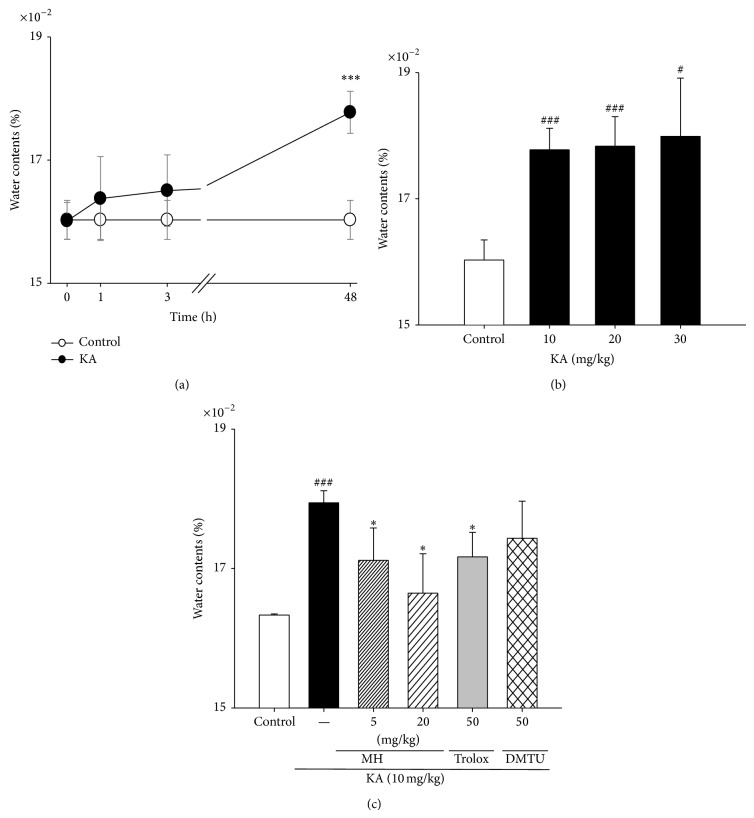
Changes and protective effects of MH, scavengers on the brain water content in KA-induced mice. (a) Male mice were grouped (*n* = 5 or 6/group). Seizures were induced by KA injection (10 mg/kg, i.p.); the mice in the saline group received an equal volume of 0.9% NaCl. Water contents were measured 0, 1, 3, and 48 h after KA or control injection. (b) Male mice were grouped (*n* = 5 or 6/group). Seizures were induced by KA injection (10–30 mg/kg, i.p.); the mice in the control group received an equal volume of 0.9% NaCl. (c) Male mice were grouped (*n* = 5 or 6/group) and pretreated with MH (5–20 mg/mouse, i.p.), trolox (50 mg/kg, i.p.), DMTU (50 mg/kg, i.p.), or NaCl (0.9%). Fifty minutes after MH or saline pretreatment, seizures in the KA and MH + KA groups were induced by KA injection (10 mg/kg, i.p.); the mice in the control group received an equal volume of 0.9% NaCl. The water contents of brain tissue was detected by measurement of the ratio of brain weight and body weight. The percentage of water content was calculated as [brain weight/bodyweight] × 100%. All weight data are presented as means ± SE. ^#^ *P* < 0.05,^###^ *P* < 0.001 versus control group. ^*^ *P* < 0.05, ^***^ *P* < 0.001 versus KA group.

To measure brain edema, we evaluated the brain water content at 48 h after i.p. treatment. As shown in Figure 2, there was a significant increase in brain water content in the KA group compared to the control group that was dependent on administration dose and time. The amount of dye extravasation in brain was markedly increased compared to the control brain at 48 h after KA treatment (17.77 ± 0.34 × 10^−2^ versus 16.02 ± 0.31 × 10^−2^). In contrast, the instillation of the control solution had no effect on the increase in water contents. Moreover, pretreatment with MH and KA resulted in a decrease in brain water content compared to the administration of KA alone (*P* < 0.05). The highest dose of the MH used in this study (20 mg/kg) inhibited the increase in water content to a degree similar to that achieved by scavengers. Scavenger treatment was associated with a significant decrease in brain weight/body weight ratios.

### 3.2. Changes in KA-Induced Brain Barrier Permeability due to MH

**Figure 3:**
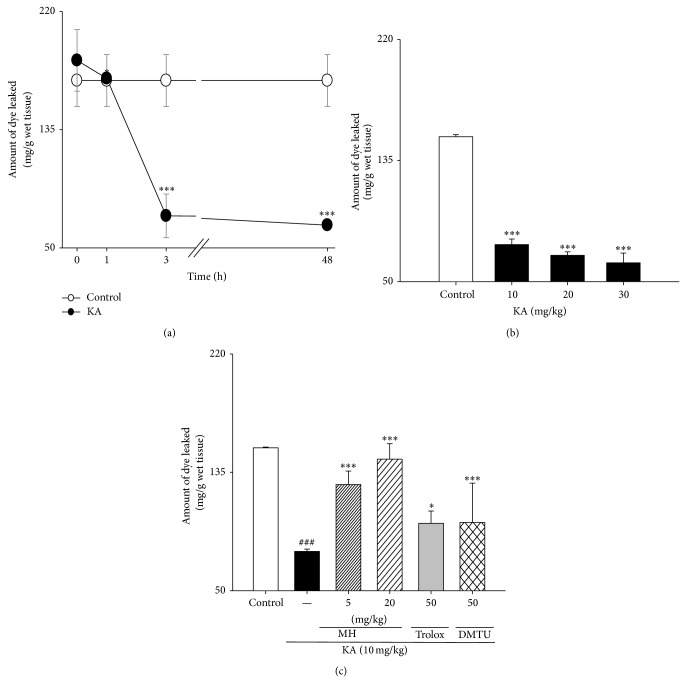
Changes and ameliorative effect of MH, scavengers on the BBB permeability in KA-induced mice. (a) Male mice were grouped (*n* = 5 or 6/group). Seizures were induced by KA injection (10 mg/kg, i.p.); the mice in the control group received an equal volume of 0.9% NaCl. Permeability was measured colorimetrically 0, 1, 3, and 48 h after KA or control injection. (b) Male mice were grouped (*n* = 5 or 6/group). Seizures were induced by KA injection (10–30 mg/kg, i.p.); the mice in the control group received an equal volume of 0.9% NaCl. (c) Male mice were grouped (*n* = 5 or 6/group) and pretreated (i.p. injection) with MH (5–20 mg/kg, i.p.), trolox (50 mg/kg, i.p.), DMTU (50 mg/kg, i.p.), or NaCl (0.9%). Fifty minutes after MH or control pretreatment, seizures in the KA and MH + KA groups were induced by KA injection (10 mg/kg, i.p.); the mice in the control group received an equal volume of 0.9% NaCl. EBD (100 mg/kg, i.v) was injected 5 minutes prior to KA administration. Two days after the KA administration, dye in the brain tissue was extracted and determined colorimetrically. All data are presented as means ± SE. ^###^ *P* < 0.001 versus control group. ^*^ *P* < 0.05, ^***^ *P* < 0.001 versus KA group.

EBD uptake after the i.p. administration of KA was measured to evaluate the loss of barrier integrity due to KA. The results of the EBD uptake studies are shown in Figure 3. The dye uptake in the mice that were given only saline prior to KA injection was 170.28 ± 18.63 mg/g wet tissue. The increase in barrier permeability was largely reversed within 3 hours. KA significantly inhibited the uptake of EB to 73.02 ± 15.65 mg/g wet tissue compared to the control, while EB uptake remained unaltered until 48 hours (66.32 ± 3.15 mg/g wet tissue). KA produced a dose-related inhibition of dye extravasation. In contrast, MH pretreatment significantly reversed the KA-elicited EBD permeability in the brain; the levels reached 126.28 ± 17.07 and 144.45 ± 27.75 mg/g wet tissue in the KA + MH 5 group and the KA + MH 20 group, respectively (Figures 3(a) and 3(c)). Similar results were obtained after the administration of DMTU or trolox, which are scavengers, rather than MH (Figure 3(c)). Taken together, these findings demonstrate that pretreatment with MH increased the KA-elicited decrease in the permeability.

### 3.3. MH Protected against KA-Induced Excitotoxicity in Mice

**Figure 4:**
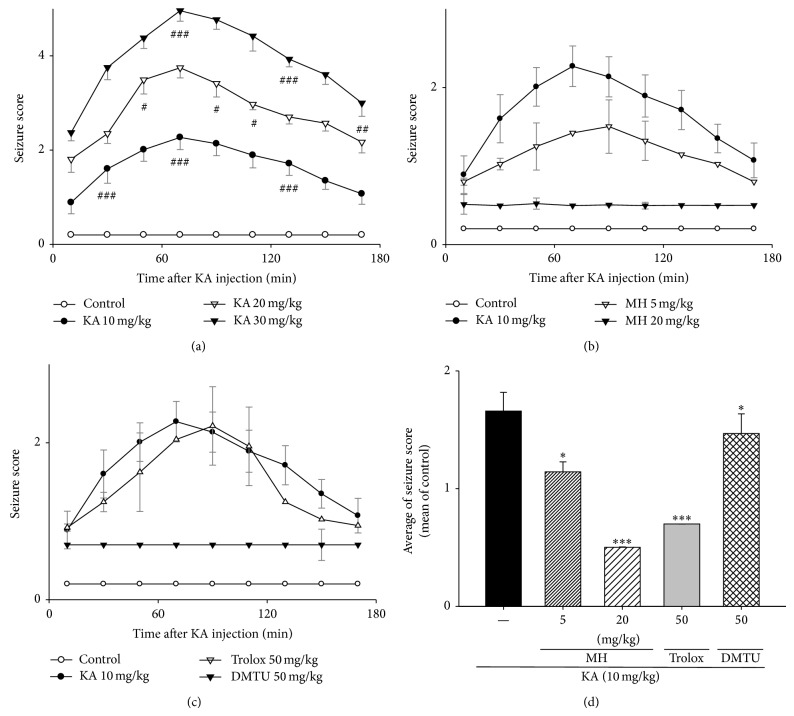
Effect of MH, scavengers on KA-induced convulsion in mice. Seizure scores were rated according to the scale devised by Racine. Seizures were induced by KA injection (10–30 mg/kg, i.p.); the mice in the control group received an equal volume of 0.9% NaCl. Male mice were grouped (*n* = 5 or 6/group) and pretreated (i.p. injection) with MH (5–20 mg/kg), scavengers such as trolox (50 mg/kg, i.p.) and DMTU (50 mg/kg, i.p.), or NaCl (0.9%). Fifty minutes after the MH or control pretreatment, seizures in mice in the KA, scavengers + KA, and MH + KA groups were induced by KA injection (10 mg/kg, i.p.); the mice in the control group received an equal volume of 0.9% NaCl. The preconvulsive behavior was scored within 3 h after KA injection. Scores at various time points after seizure induction were analyzed by two independent observers, after which they were averaged and statistically compared by the Holm-Sidak test. All data are presented as means ± SE. ^#^ *P* < 0.05, ^##^ *P* < 0.01, ^###^ *P* < 0.001 versus control group. ^*^ *P* < 0.05, ^***^ *P* < 0.001 versus KA group.

Mice were pretreated with MH (5–20 mg/kg, i.p.) to evaluate the protection of MH against KA-induced excitotoxicity. Equal volumes of 0.9% NaCl solution were administered to control mice in the same manner. Fifty minutes after the MH or saline pretreatment, KA (10–30 mg/kg) or 0.9% saline was injected. The convulsive behaviors were scored for 3 h after KA injection. Compared with the saline group in which no neurologic anomalies were observed following saline induction, the convulsive behaviors of the KA-treated mice initially occurred within 30 min; the seizure scores reached 3 after 1 h and substantially increased to 4-5 within 3 h following KA injection (Figure 4(a)). Delayed seizure onsets and decreased seizure scores were observed in the mice that were administered MH and the mice that received the scavengers trolox (50 mg/kg, i.p.) or DMTU (50 mg/kg, i.p.) (Figures 4(b) and 4(c)). The maximal seizure values were reached after 1.30–2 h; thereafter, the seizure scores gradually decreased. As shown in Figure 4(d), we arranged the average seizure scores at each time point. Significant decreases in the average of seizure scores were observed in the mice that were administered MH or the scavengers trolox (50 mg/kg, i.p.) or DMTU (50 mg/kg, i.p). Taken together, these results indicate that MH may alleviate KA-induced status epilepticus.

### 3.4. Inhibition of Hippocampal MDA Levels by MH

**Figure 5:**
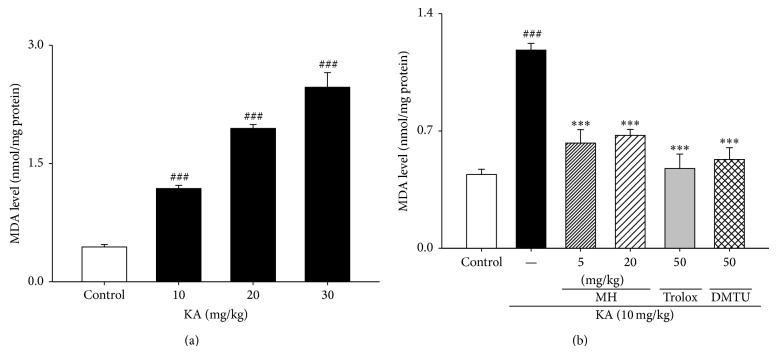
Effect of MH, scavengers on the MDA levels in KA-treated hippocampal tissue homogenates. Male mice were grouped (*n* = 5 or 6/group) and pretreated (i.p. injection) with MH (5–20 mg/kg), scavengers such as trolox (50 mg/kg, i.p.) or DMTU (50 mg/kg, i.p.), or NaCl (0.9%). Fifty minutes after the MH or saline pretreatment, seizures in the KA, scavengers + KA, and MH + KA groups were induced by KA injection (10 mg/kg, i.p.); the mice in the control group received an equal volume of 0.9% NaCl. All data are presented as means ± SE. ^###^ *P* < 0.001 versus control group. ^***^ *P* < 0.001 versus KA group.

The mechanism underlying these findings may be mediated by reactive oxygen scavenging. Free radicals may be one of the major causes of excitotoxic lesions. Therefore, we estimated free radical generation using a TBARS assay. TBARS levels are an indicator of lipid peroxidation and were significantly and dose-dependently increased in the hippocampi of the mice that were treated with KA compared to the control group that did not receive the stressor agent (Figure 5(a)). However, pretreatment with the 5 and 20 mg/kg doses of MH significantly prevented the KA-induced increase in TBARS levels. The animals that were treated only with either dose of MH exhibited no alterations in TBARS levels (data not shown).

### 3.5. Alterations in Hippocampal Cld-5 Protein Levels due to MH

**Figure 6:**
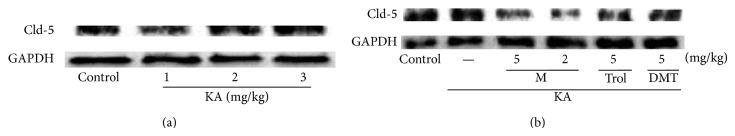
Effect of MH and scavengers on cld-5 levels in KA-treated hippocampal neuron. Immunoblots of lysed mice hippocampus 2 days following administration of MH or KA are shown. Neurons were exposed to KA at concentrations of 0, 10, 20, and 30 mg/kg for 48 h. Neurons were exposed to 10 mg/kg of KA at 0.5–1 h after 5–20 mg/kg of MH, trolox (50 mg/kg, i.p.), and DMTU (50 mg/kg, i.p.) pretreatment. GAPDH levels were measured to confirm equal protein loading.

Cld-5 can be the critical transmembrane protein that determines the barrier properties. Cld-5 tightens the paracellular cleft. As a further test, we investigated the alterations in the hippocampal tissues that were induced by KA (Figure 6). The expression of Cld-5 was dose-dependently induced by KA. Moreover, slight changes in Cld-5 expression levels were observed following the trolox and DMTU treatment. These data suggest that MH may potentially inhibit KA-induced barrier dysfunction* via* redox sensitive repair, which may be important for the physiological activity of the barrier.

### 3.6. Effect of MH and the Scavengers on KA-Induced Neuronal Loss, Oxidative Stress, and [Ca^2+^]_*i*_ Influx in Primary Cultured Hippocampal Cells

**Figure 7:**
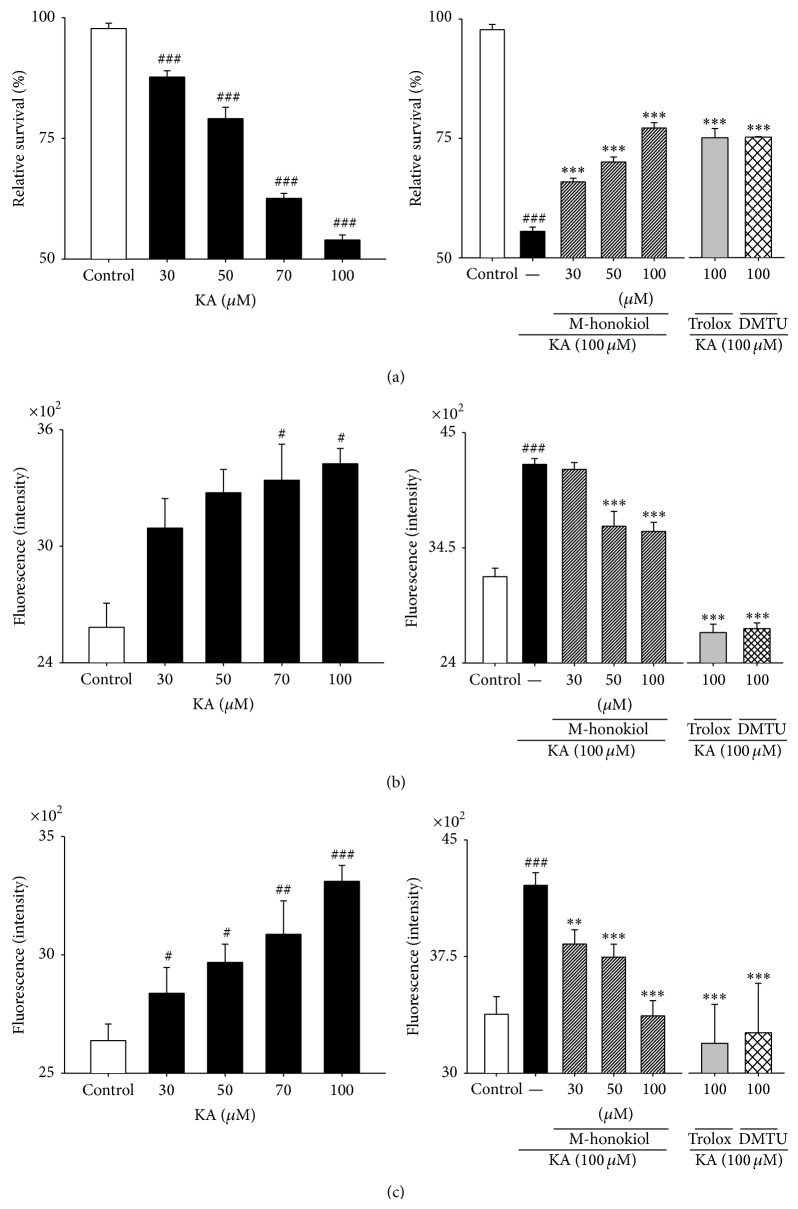
Effect of MH, scavengers on KA-induced neuronal loss and Oxidative stress and [Ca^2+^]_*i*_ influx in primary cultured hippocampal cells. Concentration data for KA-induced toxicity in primary cultured hippocampal neurons. Examination of the dose effect of KA on neuronal viability by the MTT assay. Neurons were exposed to KA at concentrations of 30, 50, and 100 *μ*M for 48 h. Cell viability at 48 h after KA exposure was measured by the MTT assay. Examination of the dose effect of KA on neuronal ROS level and [Ca^2+^]_*i*_ the DCFH-DA and Ca^2+^ indicator with fura-4 assay. All data are presented as means ± SE. ^###^ *P* < 0.001 versus control group. ^*^ *P* < 0.05, ^**^ *P* < 0.01, ^***^ *P* < 0.001 versus KA group.

The cell death of primary hippocampal neurons was assessed with MTT assays to evaluate the protective capabilities of MH. Neurons were exposed to KA at concentrations of 0, 30, 50, 70, and 100 *μ*M for 48 h (Figure 7(a)).  Figure 7 shows that cell death was rapid; growth was inhibited to 55.5 ± 2.2% of the control levels 48 h after KA exposure. When the cells were exposed to 100 *μ*M KA, significant cell death occurred after 48 h. Exposure of the hippocampal neurons to KA (100 *μ*M) for 48 h elicited a significant decrease in cell survival, whereas KA-induced neuronal loss was inhibited by 65.8 ± 2.0% and 77.1 ± 4.0% following the addition of 30 and 100 *μ*M MH, respectively (Figure 7(a)). Moreover, inhibition of cell death to 75.0 ± 3.3% and 75.2 ± 0.1% occurred following exposure to 100 *μ*M of trolox and DMTU, respectively (Figure 7). These results indicate that MH protected the neurons against KA-induced cytotoxicity. Because KA induces oxidative damage in cultured murine neurons, we examined whether MH affected the 100 *μ*M KA-induced changes in ROS levels in primary hippocampal neurons using the DCFH-DA assay. Low levels of ROS were found in the controls (3186 ± 230 fluorescence intensity), and these values were considered physiological. In contrast, a significant increase in ROS concentration was observed (4209 ± 187 fluorescence intensity) following treatment with 100 *μ*M KA for 48 h. Furthermore, as shown in Figure 7(b), the ROS levels were 4163 ± 96 and 3598 ± 204 intensity following the 30 and 100 *μ*M doses of MH, respectively. Trolox and DMTU also induced inhibitions of ROS production at the high doses of 100 *μ*M (Figure 7(b)). Taken together, these results indicate that treatment with 100 *μ*M KA elevated ROS production and that pretreatment with 30 to 100 *μ*M MH significantly decreased ROS production. Regarding oxidative glutamate toxicity, a 100-fold increase in intracellular ROS results in an elevation of cytosolic Ca^2+^ that precedes cell death. To investigate the mechanism by which MH protected against KA-induced neurotoxicity, we examined whether MH could inhibit the KA-induced elevation in intracellular [Ca^2+^]_*i*_ in cultured hippocampal neurons. We measured [Ca^2+^]_*i*_ levels using the Ca^2+^ indicator fura-4. As shown in Figure 7, the 100 *μ*M KA treatment led to a significant elevation of [Ca^2+^]_*i*_ (data not shown). The percentages of the inhibitions of the elevation of [Ca^2+^]_*i*_ due to MH in the hippocampal neurons were 8.9%, 11%, and 20% at the MH doses of 30, 50, and 100 *μ*M, respectively, the levels of the normal controls were taken as 100% (Figure 7(c)). Thus, the MH treatments in the range of 30 to 100 *μ*M dose-dependently inhibited the KA-induced elevations in [Ca^2+^]_*i*_. However, DMTU was strongly suppressed following treatment with trolox. These results indicate that the treatment of the cultured hippocampal neurons with 100 *μ*M KA elevated [Ca^2+^]_*i*_ and that this effect was attenuated by MH.

### 3.7. Reduction of DPPH Radical Activity by MH

The activity of MH is generally attributed to its antioxidative efficacy. To identify the redox potential of MH, the reduction of the DPPH radicals was analyzed using a spectrophotometric method. Figure 6 shows that MH scavenged the DPPH radicals in a dose-dependent manner and that the IC_50_ of MH was approximately 52.4 mM (Figure 6). Trolox was used as a positive control and found to scavenge 100% of the DPPH radicals at 0.25 mg/mL.

### 3.8. ^•^OH Reduction Activity of MH

The ^•^OH generated by the Fenton reaction system was trapped by DMPO, and this trapped ^•^OH was detected with an ESR spectrometer. As shown in Figure 9(a), the typical 1 : 2 : 2 : 1 ESR signal of the DMPO/^•^OH adduct (*A*N =* A*H = 14.4 G) was observed. Each spectrum was obtained 15 min after the initiation of the Fenton reaction. MH inhibited the Fenton reaction by reacting with ^•^OH. Additionally, the DMPO/^•^OH adduct signal gradually decreased over time. The decay rate exhibited approximately pseudo-first-order kinetics over the period of measurement, and the half-life of the DMPO/^•^OH signal was estimated to be 10.24 min. The activities of DMTU, trolox, and MH were 5.4, 7.65, and 9.65 min, respectively. The rate constant of the reaction of MH with ^•^OH (3.4 × 10^9^ M^−1^ *·*S^−1^) was calculated from the competition with the spin trap DMPO and found to be close to the reactivity of trolox (3.5 × 10^9^ M^−1^ *·*S^−1^) (Figure 9(b)). The initial velocity of the signal decay rate of MH seemed to be slightly faster than that of trolox.

## 4. Discussion

Our results clearly demonstrated that MH, a principal ingredient of the bark of* M. officinalis, *had significant antioxidant/neuroprotective effects against KA-induced excitotoxicity in mice. The cytological, biochemical, and behavioral results agreed with the fact that MH inhibited seizures* in vivo *and inhibited edema and permeability variations. We found that MH reduced the MDA and Cld-5 levels in the hippocampal tissue. Moreover, MH was found to possess DPPH radical and ^•^OH reduction activities.

**Figure 8:**
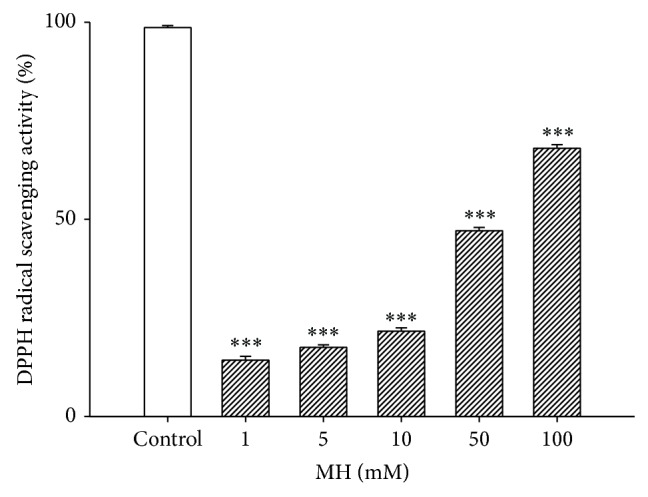
DPPH radical-scavenging activity of MH. 1–100 mM of MH was used. Trolox, as a positive control, scavenged 100% of the DPPH radical at 0.25 mg/mL. All data are presented as means ± SE. ^***^ *P* < 0.001 versus control group.

**Figure 9:**
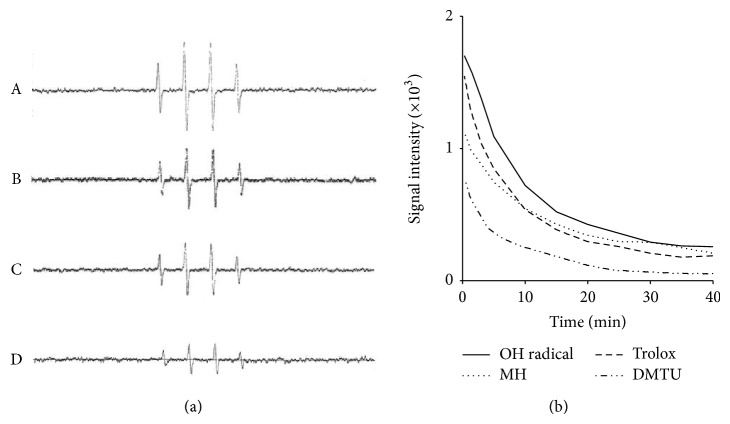
Reduction activity of MH on ^•^OH. (a) ESR spectra of DMPO/^•^OH adduct were generated in a Fenton Reaction System. The solutions with a final volume of 0.1 mL contained 2 mM ferrous sulfate, 2 mM H_2_O_2_, and 100 mM phosphate buffer (pH 7.2). Reactions were started by addition of ferrous ammonium sulfate (2 mM final concentration) and the steady-state ESR were recorded at 15 min after the Fenton Reaction. Line A; DMPO/^•^OH adduct, B; MH (1 mg/mL), C; trolox (1 mM), and D; DMTU (1 mM). (b) Time course of ^•^OH degradation induced by Fenton Reaction System. DMPO/^•^OH adduct, MH (1 mg/mL), trolox (1 mM), and DMTU (1 mM).

KA is a specific agonist of the KA receptor and a selective ionotropic glutamate agonist. Glutamate toxicity is the major contributor to pathological cell death within the nervous system and appears to be mediated by ROS [49]. Excitotoxicity is thought to play an important role in the neural damage that occurs in pathological conditions such as trauma, stroke, epilepsy, and hypoglycemia. Under such pathological conditions, the excess release of L-glutamic acid and other EAAs can lead to excitotoxic lesions in the brain that result from the overexcitation of nerve cells [50]. Behavioral activity can provide strong evidence that antioxidants or free radical scavengers are capable of counteracting the neuronal damage induced by KA (Figure 4). Schulz et al. provided direct* in vivo* evidence that KA-induced neuronal damage is mediated by free radicals. Our DPPH and ESR data indicate that MH has radical reduction activity (Figures 8 and 9).

The BBB is intimately interconnected with the causes and effects of and treatments for seizures. The endothelium is a semipermeable barrier that lines the vasculature and regulates fluid and solute exchange between the blood and the interstitial space. There are two pathways that allow solutes to traverse the endothelium, the transcellular and paracellular pathways. The transcellular pathway, also known as the transcytotic pathway, is defined by the caveolae-dependent vesicle-mediated transport of macromolecules, across the endothelial barrier. The transcellular route crosses the apical and basal cell membranes and is primarily mediated by channels, carriers, pumps, and vesicles. In contrast, the paracellular route passes through the intercellular and lateral spaces between cells that are in contact and is mediated by the tight junctions (TJs). As shown in Figure 3, MH increased the permeability of the BBB following KA-induced neurotoxicity in mice. This increase in permeability may account for the superior antioxidant activity. It is well known that KA induces free radicals [51, 52]. Free radicals actually inhibit the sodium pump [53]. Potassium ions are crucial for normal action potential generation in all excitable tissue. Another possibility is related to the facts that MH is a small molecule and a dimer of allyl-phenol. The ortho-allyl group may potentially form a six-member ring after the absorption of the hydroxyl group. Additionally, phenolic compounds are commonly found in plants and have been reported to have multiple biological effects that include antioxidant activity [54–56]. Finally, lipid solubility has long been recognized as an important factor in diffusion across biological membranes; this is partially due to hydrogen bonding affinity, which is key factor that determines the rate at which a drug passively crosses the BBB. The presence of hydroxyl groups on peptides tends to promote hydrogen bonding with solvating water, which leads to a concomitant decrease in the partition coefficient (i.e., the lipophilicity) and a subsequent decrease in membrane permeability [57]. Accordingly, MH may enhance radical scavenging in lipid and hydrophobic environments, which may be important for the physiological activity of the barrier. To assess the impaired barrier permeability for EAAs, we investigated the alterations in endothelial tight junctional protein expression. The barrier properties are primarily determined by endothelial junctional complexes that consist of TJs and adherens junctions (AJ). In general, TJs seal the interendothelial cleft to form a continuous blood vessel, while the AJs are important for initiating and maintaining endothelial cell-cell contact [58, 59]. The molecular architecture of the TJ has been recently reviewed in detail [60, 61]. Clds are the principal barrier-forming proteins. Clds have two functional subcategories: (1) Clds that specifically increase paracellular permeability through the formation of paracellular channels (pore forming Clds), and (2) Clds that are generally reduce paracellular permeability (sealing Clds) [62]. Brain endothelial cells possess Cld-5, Cld-12, and possibly other Clds. Cld-5 is a critical component of the maintenance of the barrier [63]. Cld-5 is responsible for limiting paracellular ion movement selectively, which produces the high ER of the barrier [64, 65]. Some Clds can also regulate charge selectivity by acting as electrostatic barriers to either anions or cations. Alternatively, the high ER or low conductance of the potential paracellular pathway emphasizes the extreme effectiveness of the tight junctions in occluding this pathway by effectively reducing the movement of ions.

Brain edema is commonly observed in conditions of impaired BBB function. The early process of vasogenic edema formation may also initiate seizures. Based on our edema results (Figure 2), we speculate that MH may reduce edema formation early in epilepsy by controlling the rate of ion transport across the barrier. However, many questions remain concerning the mechanisms that are responsible for barrier ion transport and how they are altered by epilepsy.

Additionally, we quantitatively estimated ^•^OH levels to examine the rate constants of the interaction of MH with ^•^OH. In this work, we studied the reactions of MH with radicals using the ESR technique. As shown in Figure 9, the DMPO/^•^OH adduct was suppressed by the presence of MH or scavengers. MH reduced ^•^OH at relatively low concentrations (1 mg/mL). The DMPO/^•^OH adduct occurred at approximately 10.24 min, whereas those for DMTU, MH, and trolox occurred at approximately 5.4, 7.65, and 9.65 min, respectively. Generally, the second-order rate constant for DMPO/^•^OH (3.4 × 10^9^ M^−1^ *·*S^−1^) is estimated with mannitol as a competitive standard [k (^•^OH  + mannitol) = 2.7 × 10^9^ M^−1^ *·*S^−1^]. The rate constant of the reaction of MH with ^•^OH (4 × 10^9^ M^−1^ *·*S^−1^) was calculated from the competition with the spin trap DMPO and was found to be close to the reactivity of trolox (Figure 9(b)). The initial velocity of the signal decay rate for MH seemed to be slightly faster than that of trolox. In summary, MH efficiently reduced DPPH (IC_50_, 52.4 mM) and ^•^OH with a rate constant of 4 × 10^9^ M^−1^ *·*S^−1^, which is close to that for the reactivity of trolox, which is a vitamin E analog.

## 5. Conclusion

These results suggest that MH may enhance redox scavenging mechanisms in lipid and hydrophobic environments, which may be important for the physiological activity of the barrier. Thus, MH can be a useful agent against redox-associated development or the progression of epilepsy.
